# *Cryptosporidium cuniculus* - new records in human and kangaroo in Australia

**DOI:** 10.1186/s13071-014-0492-8

**Published:** 2014-10-30

**Authors:** Anson V Koehler, Margaret J Whipp, Shane R Haydon, Robin B Gasser

**Affiliations:** Faculty of Veterinary and Agricultural Science, The University of Melbourne, Parkville, Victoria 3010 Australia; Microbiological Diagnostic Unit, Public Health Laboratory, Doherty Institute, The University of Melbourne, Parkville, Victoria 3010 Australia; Melbourne Water Corporation, Docklands, Victoria 3008 Australia

**Keywords:** *Cryptosporidium cuniculus*, Australia, Human, Kangaroo, Novel genotype

## Abstract

**Background:**

To date, *Cryptosporidium cuniculus* has been found exclusively in rabbits and humans. The present study provides the first published molecular evidence for *C. cuniculus* in an Australian human patient as well as a kangaroo.

**Findings:**

Using PCR-based sequencing of regions in the *actin*, 60 kDa glycoprotein (*gp60*) and small subunit of ribosomal RNA (*SSU*) genes, we identified a new and unique *C. cuniculus* genotype (akin to VbA25) from a human, and *C. cuniculus* genotype VbA26 from an Eastern grey kangaroo (*Macropus giganteus*) in Australia.

**Conclusions:**

The characterisation of these genotypes raises questions as to their potential to infect humans and/or other animals in Australia, given that *C. cuniculus* has been reported to cause cryptosporidiosis outbreaks in Europe.

## Background

*Cryptosporidium* is a genus of apicomplexan protozoans that infect humans and other animal hosts, and frequently cause enteritis and associated diarrhoea [1]. Most infections of humans are attributed to *C. hominis* and *C. parvum*, but *C. canis*, *C. felis* and *C. meleagridis*, which usually infect dogs, cats and birds, respectively, are also zoonotic [2]. Interestingly, *C. cuniculus*, usually a parasite of rabbits, is genetically closely related to *C. hominis* and *C. parvum*, and has been responsible for outbreaks of cryptosporidiosis in humans in the UK [3-5]. In the first recorded outbreak, the infection source was water from a tank contaminated by *C. cuniculus*-infected European rabbit (*Oryctolagus cuniculus*), and the protist was identified with the aid of molecular tools [6].

Using the sequences of small subunit of ribosomal RNA (*SSU*), *actin* and/or *Cryptosporidium* oocyst wall protein (*COWP*) genes, *C. cuniculus* can be distinguished genetically from the closely related species *C. hominis*. In addition, *C. cuniculus* can be readily identified based on the sequence of the 60 kDa glycoprotein (*gp60*) gene. Using this locus, two distinct clades, designated Va and Vb, can be classified according to the number of TCA (serine)-tandem repeats in the microsatellite region (e.g., VaA26 = clade Va, with 26 TCA repeats) [3]. Although there is no evidence to suggest that either clade is strictly host-specific, most cases described in humans relate to clade Va [7].

Since the first UK outbreak [3], there has been increasing interest in *C. cuniculus* due to the zoonotic threat that it poses [6], and this pathogen has been identified in humans and/or rabbits mainly in the UK and China [3,7-10], with some reports from other countries including the Czech Republic, France and Nigeria [11-13]. In addition, a recent outbreak was associated with the deaths of 300 rabbits in Poland [14]. Although *C. cuniculus* has been recorded in rabbits in south-eastern Australia [15,16], there is no published report of this pathogen from humans or other host species in Australia. Here, we report *C. cuniculus* genotypes originating from an Australian human patient and, for the first time, from an Eastern grey kangaroo (*Macropus giganteus*).

## Methods

A faecal sample (collected in 2009) from a human known to be clinically affected by cryptosporidiosis was provided to us (anonymously) by the Microbiology Diagnostic Unit of the Public Health Laboratory (MDU/PHL) of the University of Melbourne. Faecal DNA was isolated following treatment with 10% polyvinyl-polypyrrolidone, using the standard QIAamp DNA Mini Kit (Qiagen, Germany) protocol. In addition, a faecal sample from *M. giganteus* was collected during a surveillance campaign of waterborne pathogens in wildlife within the drinking water catchment areas of Melbourne Water (www.melbournewater.com.au) in Victoria, Australia [16]. This faecal sample was collected in August 2013 in the Yan Yean catchment (-37.619704; 144.901997) [16]; the species of kangaroo host was initially established by sight, according to Triggs [17], and then confirmed using an established PCR-based method (unpublished) for the sequencing of a mitochondrial cytochrome oxidase *b* gene region of *M. giganteus* from faecal DNA isolated using a kit (PowerSoil, Mo-Bio, California). Subsequently, regions of the *actin*, *gp60* (two regions) and/or *SSU* genes of *Cryptosporidium* were amplified individually from each of the two faecal DNA samples by PCR and then sequenced as described previously [18]. Nucleotide sequences were deposited in the GenBank database [GenBank: KM366138-KM366142].

## Findings

**Figure 1:**
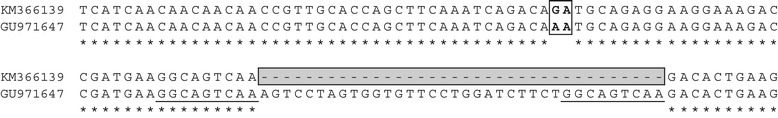
**Sequence alignment.** An alignment of the sequence of part of the *gp60* gene (derived from a *Cryptosporidium cuniculus* genotypes from a human) [GenBank: KM366139] with its closest sequence match in the GenBank database [GenBank: GU971647] (*C. cuniculus* from human). Bold-type indicates a variable region of two bases. The deletion is indicated in grey (36 positions). The repeat regions before and after the deletion are underlined. Identical nucleotides are indicated with an asterisk.

*C. cuniculus* was identified in the human faecal DNA sample based on the sequences of amplicons derived from the *SSU* [GenBank: KM366141] (252 bp), *actin* [GenBank: KM366138] (782 bp) and *gp60* [GenBank: KM366139] (243 bp) gene regions; no sequence differences were detected in the *SSU* or *actin* regions compared with published sequences for *C. cuniculus* [GenBank: EU437413 and GU327783]. Based on the tandem repeat region in *gp60*, the parasite detected was classified as *C. cuniculus* VbA25. Of interest was a 36 bp deletion determined by comparison with a *gp60* reference sequence [GenBank:KM366139] (Figure 1). This deletion was confirmed by independent sequencing of this region using two independent sets of primers to *gp60* and did not result in a frame shift in the coding region (*c.f.* Figure 1). With the exception of this deletion and a single nucleotide substitution (G→A) (Figure 1), the *gp60* sequence (representing VbA25) was the same as that of a reference sequence [GenBank: GU971647] from a human clinical case in the UK [8]; it was also 98% similar to another *C. cuniculus* sequence from a rabbit in Australia [GenBank: KC283003] (also classified as VbA25), but contained an additional ‘ACA’ repeat following the characteristic serine tandem repeat region.

*C. cuniculus* was also identified in the faecal sample from a *M. giganteus* individual from the Yan Yean catchment based on the *SSU* [GenBank: KM366142] (557 bp) and *gp60* [GenBank: KM366140] (282 bp) sequences from amplicons produced. The *gp60* sequence (VbA26) was similar to that from *C. cuniculus* (VbA25; with an additional serine repeat) [GenBank: KC283003] from the same catchment (2011), and identical to sequences [GenBank: HM852433 and KC283004] originating from *C. cuniculus* from the same lagomorph species from another Melbourne Water catchment (Sugar Loaf; -37.681999; 145.293841) in 2010.

To date, *C. cuniculus* has been found exclusively in rabbits and humans [6,7,14]. Here, we provide molecular evidence for a new *C. cuniculus* genotype in a human in Australia and, for the first time, for the occurrence of *C. cuniculus* in a kangaroo. In Victoria, Australia, as part of an ongoing pathogen detection program, in which we have molecularly tested >1500 faecal samples from kangaroos and wallabies ([16]; Koehler *et al*. unpublished data), this is the first instance of *C. cuniculus* being detected in the faeces from any macropodid host. The presence of *C. cuniculus* DNA in the faecal sample from the *M. giganteus* individual indicates, but does not prove, that this macropod was infected with this pathogen. There is a remote possibility that *C. cuniculus* oocysts passed in faeces related to pseudoparasitism [19], as a result of the kangaroo ingesting such oocysts while grazing on grass contaminated by faeces from infected rabbits. Nonetheless, given that many kangaroos graze in some of Melbourne’s water catchment areas, where rabbits are common, we would have expected to identify *C. cuniculus* much more frequently by molecular testing than one of >1500 samples tested to date. Therefore, the probability that this *M. giganteus* individual harboured a cryptosporidial infection is considered high.

The zoonotic risk of *C. cuniculus* to human health is clearly evidenced by the first outbreak in the UK in 2008, in which 29 people reported illness after drinking water contaminated with *C. cuniculus* from a rabbit [5]. In addition, recently, the dramatic effect of *C. cuniculus* on its lagomorph host was demonstrated when 300 rabbits were reported to have died from cryptosporidiosis linked to this species of *Cryptosporidium* (*c.f*. [14]). The occurrence of *C. cuniculus* genotypes in rabbits [15] and, here, in *M. giganteus* suggests that such genotypes might be able to spread to other native mammals and/or humans in Australia. Therefore, there is a need to diligently monitor *Cryptosporidium* in the vicinity of drinking water catchments (*c.f*. [16]) and in drinking water. The need for monitoring by molecular tools is particularly crucial in Victoria and other states of Australia, where rabbit populations have flourished ever since their introduction in 1859, causing a widespread degradation of natural ecosystems [20]. Such monitoring is also critical, given that people in Victoria consume unfiltered drinking water from natural catchment areas, and that currently used water treatment processes/procedures do not destroy oocysts of *Cryptosporidium* [21].

## Conclusions

Sequencing of regions in the *actin*, *gp60* and *SSU* genes has led to the identification of a new and unique *C. cuniculus* genotype (similar to VbA25 from a human); for the first time, *C. cuniculus* (genotype VbA26) has been found in an Eastern grey kangaroo (*M. giganteus*) in Australia. The characterisation of this and similar genotypes raises questions as to their potential to infect humans and/or other animals in Australia, given that *C. cuniculus* has been reported to cause cryptosporidiosis outbreaks in Europe.
